# Postural Instability in Children with ADHD Is Improved by Methylphenidate

**DOI:** 10.3389/fnins.2016.00163

**Published:** 2016-05-04

**Authors:** Maria P. Bucci, Coline Stordeur, Eric Acquaviva, Hugo Peyre, Richard Delorme

**Affiliations:** ^1^UMR 1141 Institut National de la Santé et de la Recherche Médicale—Université Paris Diderot, Robert Debré HospitalParis, France; ^2^Child and Adolescent Psychiatry Department, Robert Debré HospitalParis, France; ^3^Université Paris DiderotParis, France

**Keywords:** ADHD, children, postural control, cerebellum, sensorimotor integration

## Abstract

**HIGHLIGHTS**

- Both spatial and temporal analyses of the Center of Pressure demonstrate that children with ADHD have poorer postural control than typically developing sex-, age-, and IQ-matched children.
- Poor sensory integration in postural control could partially explained the deficits in postural stability in children with ADHD.
- MPH treatment improves postural performance in both spatial and temporal domains in children with ADHD.
- MPH improves postural control specifically when visual and proprioceptive inputs are misleading.
- Such improvement could be due to MPH effects on neurons, facilitating cerebellar processing of postural control.

The aim of this study was to examine postural control in children with ADHD and explore the effect of methylphenidate (MPH), using spatial and temporal analyses of the center of pressure (CoP). Thirty-eight children with ADHD (mean age 9.82 ± 0.37 years) and 38 sex- age- and IQ-matched children with typically development were examined. Postural stability was evaluated using the Multitest Equilibre machine (Framiral®) at inclusion and after 1 month of MPH in children with ADHD. Postural stability was assessed by recording under several conditions: with eyes open and fixed on a target, with eyes closed and with vision perturbed by optokinetic stimulation, on stable and unstable platforms. At inclusion, we observed poor spatial and temporal postural stability in children with ADHD. The spectral power index was higher in children with ADHD than in controls. Canceling time was shorter at low and medium frequencies of oscillation and longer at higher frequencies in children with ADHD. After 1 month of MPH, the surface area and mean velocity of the CoP decreased significantly under the most complex conditions (unstable platform in the absence of proprioceptive and visual inputs). The spectral power index decreased significantly after MPH while the canceling time did not change. Poor postural control in children with ADHD supports the hypothesis of cerebellar dysfunction in this disorder. Postural control could be improved by a more efficient processing of sensory inputs (a high-level process), as suggested by the decrease in spectral power index after MPH without changes in the canceling time (a low-level process).

## Introduction

Attention deficit hyperactivity disorder (ADHD) is characterized by abnormal behavioral patterns, including inattention, hyperactivity, and impulsivity. ADHD affects ~5% of children in Western countries (American Psychiatric Association, 2013). Associated with these main clinical features, children with ADHD perform worse than typical developing (TD) children in gross and fine motor control tasks as well as in the adaptation of motor control to changes in the environment (Piek et al., 1999; Wang et al., 2011; Papadopoulos et al., 2014). Similarly, poor control of balance has been reported in children with ADHD (Shum and Pang, 2009).

The motor control deficit in ADHD may be related to a deficit in the integration and processing of various sensory inputs i.e., visual and vestibular inputs (Zang et al., 2002; Wang et al., 2003). The cerebellar vermis, which plays an important role in the processing of sensory information, has a major function in postural control (Timmann and Diener, 2003). ADHD has been consistently reported to be associated with a smaller cerebellar vermis (Castellanos et al., 1996). A recent study comparing postural abilities in children with ADHD or with cerebellar lesions (astrocytoma resection) to controls (Buderath et al., 2009) revealed significantly poor postural performance in both groups of patients compared to TD controls (but this effect was milder in children with ADHD). Among children with ADHD, these deficits were most obvious under the most stringent conditions, in which proprioceptive and/or visual information was reduced or not available to stabilize posture. These findings support the hypothesis that cerebellar dysfunction could participate in the postural deficits in ADHD.

Methylphenidate (MPH) is commonly used to treat patients with ADHD (Wilens et al., 2002). Specifically, MPH increases the inhibitory ability of the prefrontal cortex, thus allowing for improved control of hyperactive and impulsive symptoms. For example, chronic MPH significantly improves performance on aspects of the Go/NoGo test, and these improvements are associated with improvements in clinical response (Coghill et al., 2007). Moreover, prefrontal cortex activation might be affected by MPH, depending on the degree of difficulty of the task (Matsuura et al., 2014). However, in addition to the prefrontal cortex, MPH also increases the activity of basal ganglia and the cerebellum in children with ADHD (Czerniak et al., 2013). For example, after MPH treatment, the neural activity of the cerebellum reaches values similar to those observed in TD children (Czerniak et al., 2013). Recently, Ivanov et al. (2014), investigated differences in the surface morphometry of the cerebellum in children with ADHD compared to TD children. This study showed that in children with ADHD, the volumes of the left anterior and the right posterior cerebellar hemispheres, in particular lobules IV, V, and VI, the left crus I and right crus II were significantly smaller with respect to controls. Interestingly, children with ADHD who were taking MPH at the time of the study showed significantly larger volumes of the left cerebellar surface (left lobules IV, V, VI, X, and right crus I and II) compared to non-treated children with ADHD. Thus, MPH could facilitate neural plasticity at the cerebellar level.

The effect of MPH in motor control in children with ADHD remains controversial. Using the Movement Assessment Battery, Flapper et al. (2006) reported an improvement in the motor abilities of children with ADHD after MPH treatment. Leitner et al. (2007) explored the quality of walking when children with ADHD were engaged on a dual task (listening to a text using a Walkman-like device), before and after MPH treatment. After MPH treatment, children with ADHD showed significantly reduced stride-time variability, suggesting that MPH could improve the function of dopaminergic networks, thus improving rhythmicity, attention, and motor control in children with ADHD. Buderath et al. (2009) also reported minor improvements in motor control in children with ADHD after MPH treatment. Jacobi-Polishook et al. (2009) explored postural stability under single- and dual-task conditions (while performing a memory-attention demanding task such as memorizing children's songs while listening to music) in children with ADHD after MPH treatment. Children showed a significant improvement in postural stability after MPH treatment only under the dual-task conditions, but not when performing a single task. These authors hypothesized that MPH treatment could improve attention to the secondary task in children with ADHD, leading to better postural stability, given that motor control became more automatic. Our group (Bucci et al., 2014) previously explored postural stability during a dual task in two independent groups of children with ADHD, with or without MPH treatment, compared to age-matched TD control children. The surface area covered by the center of pressure (CoP) was similar in the group of children with ADHD treated with MPH and in the group of TD children, but larger in children with ADHD who were not treated. Our results suggest poor stability control in children with ADHD.

However, our exploration was limited by the spatial assessment of the CoP and the inability to test postural performance in children with ADHD before and after MPH. The measurement of the CoP under dynamic postural conditions can provide insight into physiological and pathological mechanisms (Yelnik and Bonan, 2008). Also, relevant information on the dynamics of the CoP can be obtained by applying nonlinear analysis methods. The wavelet transformation method reveals deficits or changes in the dynamics of the postural control system (Bernard-Demanze et al., 2009), and could contribute to identifying which sensory systems are implicated or altered during a given postural task.

In the present study, we used both static and dynamic analyses of the CoP in order to gain insight into the characteristics and mechanisms involved in postural deficiency in children with ADHD. Next, we examined postural stability in these children after 1 month of MPH treatment. We hypothesized that children with ADHD would be globally less stable than TD children, especially in the dynamic situation when vision is perturbed and more attention is needed to reduce the postural deficit. Moreover, we expected that MPH would improve postural stability, with this effect being more significant under more complex investigative conditions.

## Materials and methods

### Subjects

Thirty-eight children with ADHD (mean age 9.82 ± 0.37 years) and 38 IQ-, sex-, and age-matched TD children were enrolled in the study at the Child and Adolescent Psychiatry Department, Robert Debré Hospital, (Paris, France). Trained child psychiatrists assessed all subjects. The diagnosis of ADHD according to DSM-5 criteria (American Psychiatric Association, 2013) was carried out using the Kiddie-SADS-EP (Goldman et al., 1998). Psychiatric comorbidities were systematically screened for during the interview. ADHD symptom severity was assessed using the ADHD Rating Scale-parental report (ADHD-RS). This scale is based on a large collection of normative data and has demonstrated reliability and discriminant validity in children and adolescents (Du Paul et al., 1998; Collett et al., 2003). Children with ADHD were also assessed using the Wechsler scale (Wechsler Intelligence Scale for Children fourth edition), the Beery-Buktenica Developmental Test of Visual-Motor Integration (VMI, Beery, 1997) and the Motor Assessment Battery for Children (MABC) (Henderson and Sugden, 1992).

Controls were directly interviewed to confirm the absence of ADHD. To be included in our study, controls also needed to have a total score ≤ 10 on the ADHD-RS (Dickson et al., 2011) and a neurological examination in the normal range. IQ in controls was estimated in two subtests, assessing verbal ability (the similarities test) and performance ability (matrix reasoning test). The score on these two tests was not significantly different between the two groups of children [*F*_(1, 74)_ = 1.02, *p* = 0.3 and *F*_(1, 74)_ = 1.45, *p* = 0.10 for the similarities and matrix reasoning tests, respectively]. The clinical characteristics of children with ADHD and controls are summarized in Table 1.

**Table 1:** **Clinical characteristics of ADHD children and control children tested**.

	**TD**	**ADHD**	**Subsample of children with ADHD treated with MPH**	
	***N* = 38**	***N* = 38**	***N*** = **26**	
CLINICAL DATA				
Age (years), mean ± SEM	9.70 ± 0.43	9.82 ± 0.37	9.87 ± 0.48	
Height (cm), mean ± SEM	133 ± 40	135 ± 31		
Weight (Kg), mean ± SEM	34.2 ± 2.5	33.3 ± 3.3		
Gender (male/female)	30/8	34/4	24/2	
ADHD-RS				
ADHD-RS total score, mean ± SEM	4 ± 0.8	31.8 ± 1.1	38.2 ± 1.1	28.2 ± 2.1^#^
ADHD-RS Inattention subscore, mean ± SEM	–	20.0 ± 0.6	19.5 ± 0.8	14.3 ± 1.0^#^
ADHD-RS Hyperactivity/Impulsivity subscore, mean ± SEM	–	18.8 ± 0.8	18.7 ± 1.0	13.9 ± 1.1^#^
MPH Dose in mg/kg, mean ± SEM	–	–	0.60 ± 0.04	
WECHSLER scale (WISC-IV) scores, mean ± sem				
Verbal comprehension subscale	–	100.1 ± 2.8	99.8 ± 3.9	
Perceptual reasoning subscale	–	94.8 ± 3.0	95.0 ± 4.1	
Working memory subscale	–	84.1 ± 3.3	85.5 ± 3.2	
Processing speed subscale	–	89.8 ± 2.4	90.2 ± 3.1	
Similarities test	10.36 ± 0.4	10.18 ± 0.5	10.1 ± 0.7	
Matrix reasoning test	10.54 ± 0.5	9.81 ± 0.4	10.2 ± 0.5	

# *ADHD-RS scores after MPH treatment*.

Twenty-six of the ADHD children (mean age 9.87 ± 0.48 years) were also examined 1 month after MPH treatment was started.

The investigation adhered to the principles of the Declaration of Helsinki and was approved by our Institutional Human Experimentation Committee (Comite de Protection des Personnes CPP, Ile de France V, Hôpital Saint-Antoine). Written informed consent was obtained from the participants' parents after the nature of the procedure had been explained.

### Postural recording

Postural performance was evaluated using the Multitest Equilibre, also called Balance Quest, from Framiral®, with a static/dynamic platform by Micromedical Technologies (www.framiral.fr). The platform consists in a force plate mounted on a translator, which allows a translation of the subject in the antero-posterior (y) or the medio-lateral direction (x). A computer-controlled mechanism allows sinusoidal displacements of 62 mm amplitude with adjustable velocities and frequencies. In our experimental conditions, the ramp mode allowed forward and backward translations of the force plate, with constant linear velocities of 0.03 and 0.07 m/s. For the sinusoidal mode, the frequency was 0.25 Hz. The displacement of the CoP was sampled at 40 and 100 Hz under static and dynamic conditions, respectively, and digitized with 16-bit precision (Ghulyan et al., 2005; Bernard-Demanze et al., 2009).

### Postural recording procedure

The child was placed on the Framiral® platform in a dark room (see for details Gouleme et al., 2014). The child was positioned with the feet aligned in parallel on the footprints, and arms hanging along the body. The platform was located in a room large enough to prevent acoustic and spatial orientation.

Postural recording was performed on both a stable (S) and an unstable (U) platform, under three different visual conditions: eyes open and fixed on a target (EO), eyes closed (EC), and eyes open but with perturbed vision (optokinetic stimulation, OPTO). The child was asked to stay as stable as possible. During the EO condition, the child had to focus on a small red light at a distance of 2.5 m. The optokinetic stimulation was performed using an optokinetic ball. The ball was projected on a wall at a distance of 2.5 m from the child's eyes and turned at an angular speed of 158° per second (Ionescu et al., 2006). The duration of each postural recording was 30 s, with a 15-s rest period between conditions to reduce the possible effects of tiredness. The order of the conditions varied randomly across children.

### Data processing

During upright standing condition, normal subjects produced smaller displacement of the CoP than subjects with poor postural control (Brandt, 2003). For example, subjects with Parkinson's disease have high displacement of the CoP, leading to instability, and falls during daily living. In our study, groups were age-matched to compare postural capabilities since postural stability improves with age during childhood (Assaiante and Amblard, 1995). Analysis in both spatial and temporal domain is described below.

### Classical analysis in spatial domain

**Figure 1:**
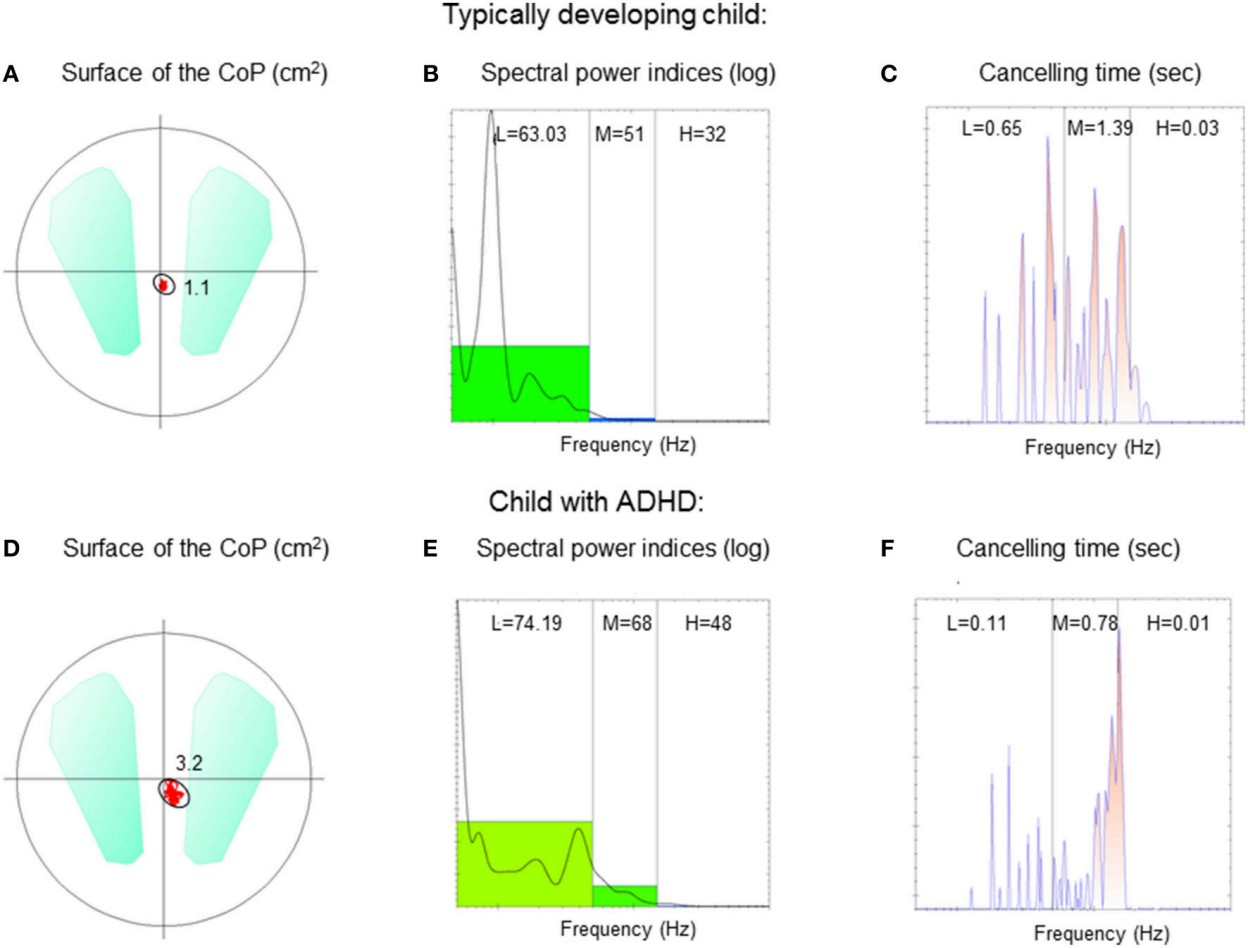
**Surface area of the CoP (cm^**2**^), spectral power indices (log), and canceling time (s), respectively for low (L), medium (M), and high (H) frequency recorded in a typically developing child (A–C) and in a child with ADHD (D–F) during the eyes open condition on stable platform**.

The surface area covered by the displacement of the CoP (cm^2^) and its mean velocity (mm/s) were analyzed in order to quantify postural performance. The surface area is an efficient measure of CoP spatial variability, corresponding to an ellipse including 90% of CoP excursions (Chiari et al., 2009). The mean velocity of the CoP is a good index of the amount of neuromuscular activity required to achieve postural control (Maki et al., 1990; Geurts et al., 1993). The mean velocity of the CoP is the mean velocity of CoP displacements over the sampled period, i.e., the sum of displacement scalars over the sampling period divided by the sampling time. Figure 1 shows an example of the surface area of the CoP recorded from both children with ADHD and typically developing (Figures 1A,D, respectively).

### Temporal analysis, wavelet transformation

We applied wavelet analysis to study the frequency of CoP displacements. A wavelet non-linear analysis using Morlet waves was applied to CoP displacements in order to elaborate a time-frequency chart of body sways (Dumistrescu and Lacour, 2006; Bernard-Demanze et al., 2009). Such analysis allows revealing temporal fluctuations in the body sway spectrum. The time-frequency plane's principle advantage is its double resolution (time and frequency). The wavelet analysis was applied on the anteroposterior sway data. From this analysis, the spectral power index (PI) and the canceling time (CT) were extracted for anteroposterior body sway and for three frequency bands (low: 0.05–0.5 Hz; medium: 0.5–1.5 Hz; high: > than 1.5 Hz). The analysis of displacements (and of associated parameters) was obtained with the software PosturoPro (Framiral, Cannes, France). The Power Indice represents the amount of energy spent during a condition and is the integral of the surface of the CoP oscillations. The canceling time is the total time during which the spectral power of the body sway (for a specific frequency band) is canceled by the postural control mechanisms. The hypothetical physiological significance of the different bands is as follows. 0–0.5 Hz: visual-vestibular (Naschner, 1979; Kohen-Raz et al., 1996); 0.5–1.5 Hz: cerebellar (Paillard et al., 2002); and 1.5 Hz: reflexive loops (Lacour et al., 2008; Bernard-Demanze et al., 2009). Power in the higher band is generally minimal in healthy subjects during quiet standing. It can nevertheless be non-negligible in elderly, in the context of postural disorders or in dynamic postural conditions (Bernard-Demanze et al., 2009). The longer the CT of a given frequency band, the better the postural control (Dumistrescu and Lacour, 2006; Bernard-Demanze et al., 2009). When the postural control system is successfully engaged, the overall entropy of the sway is reduced and the frequency's power is reduced to zero. Mechanisms involved in the canceled frequencies are not known, but the minimization of muscular effort required for controlling the sway seems one of the system's main factors. Figure 1 shows an example of PI and CT recorded from a TD child (Figures 1B,C) and a child with ADHD (Figures 1E,F).

Note that when the CoP is analyzed spatially only the behavior of the CoP over time is thus unknown and the respective role of the different sensory systems also still remains unclear. In contrast, important information on the dynamic of the CoP may be reached by applying the wavelet transformation method. Indeed, a study from Ghulyan et al. (2005) have demonstrated that a dynamic analysis of posture allows a better discrimination of the pathological effects on postural control. Yelnik and Bonan (2008) reported that temporal analysis allows us to gain insight into the physiological and pathological mechanisms underlying postural stability impairment in patients suffering from a balance disorder. According to these previous works, we made the hypothesis that longer power index associated with a shorter canceling time at a given frequency band could suggest poor postural stability most likely due to a low use of sensorial information which are believed to be associated to low, medium, and high frequencies (visuo-vestibular, cerebellar, and proprioceptive, respectively).

### Statistical analysis

Statistical analysis using two-way ANOVA was carried out in order to compare the postural control of the two groups of children under the different visual and postural conditions (EO, EC, and OPTO on static -S- and unstable supports -U-, respectively). *Post-hoc* comparisons were made with the Fischer's least significant difference test (LSD). Individual Student's *t*-tests were used to compare postural values obtained before and 1 month after MPH treatment. The effect of a factor was considered significant when the *p*-value was below 0.05.

## Results

### Children with ADHD vs. TD children

#### Static postural exploration

**Figure 2:**
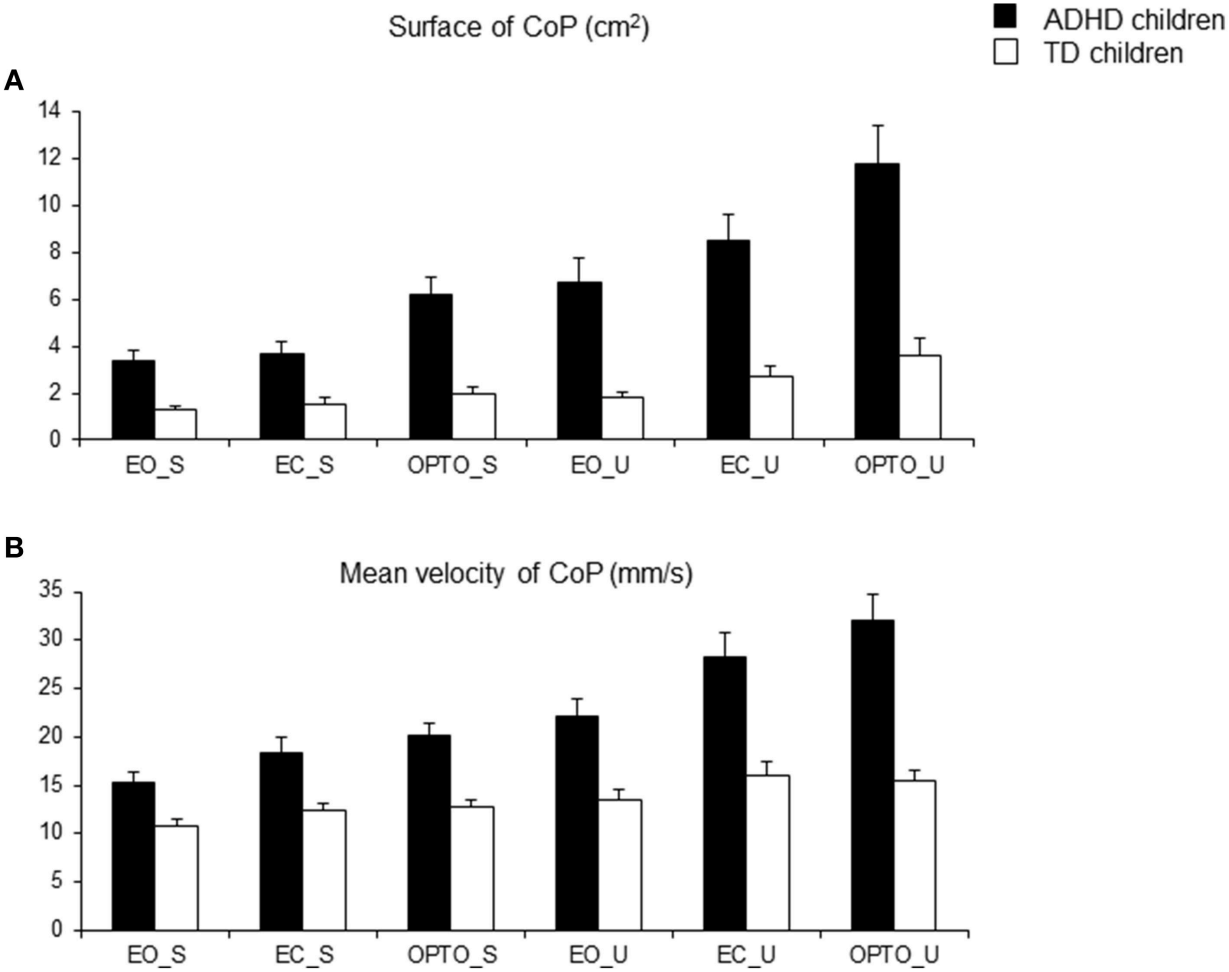
**Means and standard deviations of the surface area covered by the CoP (in cm^**2**^) (A) and the mean velocity of the CoP (mm/s) (B) in the three visual conditions (eyes open: EO; eyes closed: EC; and with vision perturbed by optokinetic stimulation: OPTO) on a stable -S- or unstable -U- platform in ADHD and control children**.

The surface area of the CoP (Figure 2A) and its mean velocity (Figure 2B) are reported in Figure 2. Independent of conditions, the surface area of the CoP in children with ADHD was systematically significantly larger than that observed in controls. ANOVA showed a significant group effect [*F*_(1, 74)_ = 31.9, *p* < 0.001], a significant postural condition effect [*F*_(1, 74)_ = 34.5, *p* < 0.001] (in both groups of children, the surface area was larger in the unstable postural condition) and a significant visual condition effect [*F*_(2, 148)_ = 16.23, *p* < 0.001]. *Post-hoc* analysis revealed that the smallest value of the surface area of the CoP was reported in the EO condition while the largest was observed in the OPTO condition (*p* < 0.001 for all).

ANOVA also showed significant differences in the mean velocity of the CoP between groups and experimental conditions (Figure 1B). Independent of condition, the mean velocity in children with ADHD was significantly higher than in TD children [*F*_(1, 74)_ = 35.33, *p* < 0.001]. There was also a significant postural condition effect [*F*_(1, 74)_ = 36.1, *p* < 0.001] (in both groups of children, the mean velocity was greater in the unstable postural condition), and a significant visual condition effect [*F*_(2, 148)_ = 17.45, *p* < 0.001]. *Post-hoc* comparisons revealed that the mean velocity of the CoP in the EO condition was lower than that reported in the EC and OPTO conditions (*p* < 0.001 for both).

To summarize, children with ADHD have poor postural control that is worsened in a dynamic environment and when visual information is perturbed or absent.

#### Temporal postural exploration

**Figure 3:**
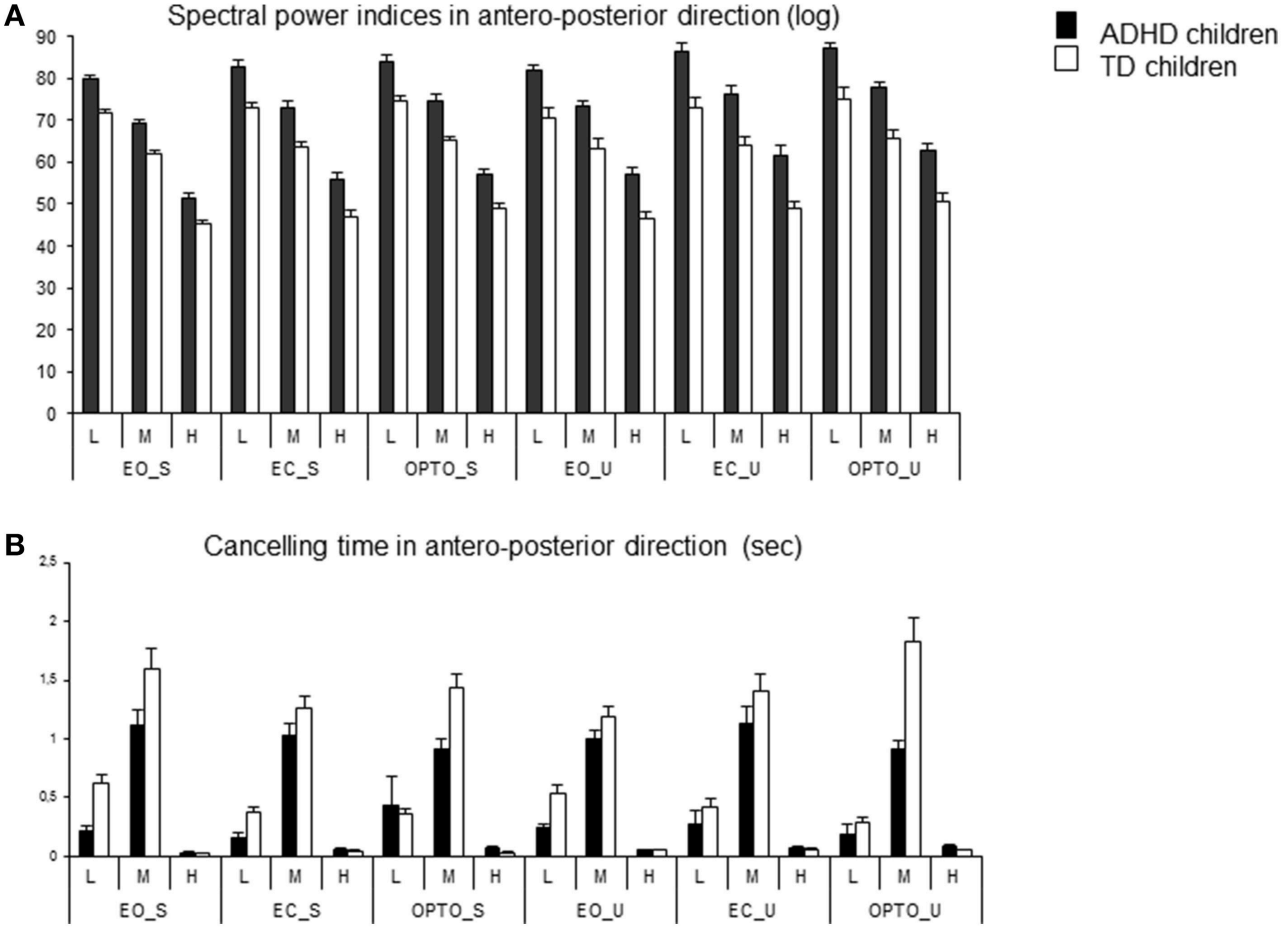
**Spectral power index (A) and canceling time (B) in the anteroposterior direction for each frequency band (L, low; M, medium; H, high) in the three visual conditions (eyes open: EO; eyes closed: EC; and with perturbed vision: OPTO) on a stable -S- or unstable -U- platform, in ADHD and control children**.

Using wavelet transformation, the spectral power index was significantly larger in children with ADHD, independent of experimental conditions (Figure 3A). ANOVA revealed a significant group effect [*F*_(1, 74)_ = 37.16, *p* < 0.001], a significant postural condition effect [*F*_(1, 74)_ = 6.12, *p* < 0.01; the spectral power index was significantly higher in the unstable postural condition], and a significant visual condition effect [*F*_(2, 148)_ = 16.33, *p* < 0.001]. *Post-hoc* comparisons showed that the spectral power index in the EO condition was smaller than that reported in the EC and OPTO conditions (*p* < 0.001 for both). ANOVA also revealed a significant frequency effect [*F*_(2, 148)_ = 1362, *p* < 0.001]. *Post-hoc* comparisons showed that the highest spectral power index was recorded for low frequencies while the lowest was recorded for high frequencies (*p* < 0.001 for all).

The canceling time in the anteroposterior direction was not different between the two groups of children examined (Figure 3B). ANOVA showed a significant frequency effect [*F*_(2, 148)_ = 247, *p* < 0.001]. The canceling time recorded for medium frequencies was significantly higher than those reported for lower and higher frequencies (*post-hoc* analysis, *p* < 0.001 for all). Interestingly, there was a significant interaction between postural and visual conditions [*F*_(2, 148)_ = 4.72, *p* < 0.001] and between groups and frequency [*F*_(2, 148)_ = 9.96, *p* < 0.001]. *Post-hoc* comparisons revealed that the canceling time was longer in the stable EO condition than in the other conditions (all *p* < 0.001). In contrast, the canceling time recorded with visual perturbation was similar in both stable and unstable conditions. Furthermore, children with ADHD showed significantly shorter canceling times than TD children for low and medium frequency bands (both *p* < 0.001) but longer canceling times for the high frequency band (*p* < 0.001).

In conclusion, both static and dynamic analyses of CoP displacement reveal differences in postural control between children with ADHD and TD children: postural stability in children with ADHD is deficient with respect to control children.

#### Impact of MPH on postural stability in children with ADHD

**Figure 4:**
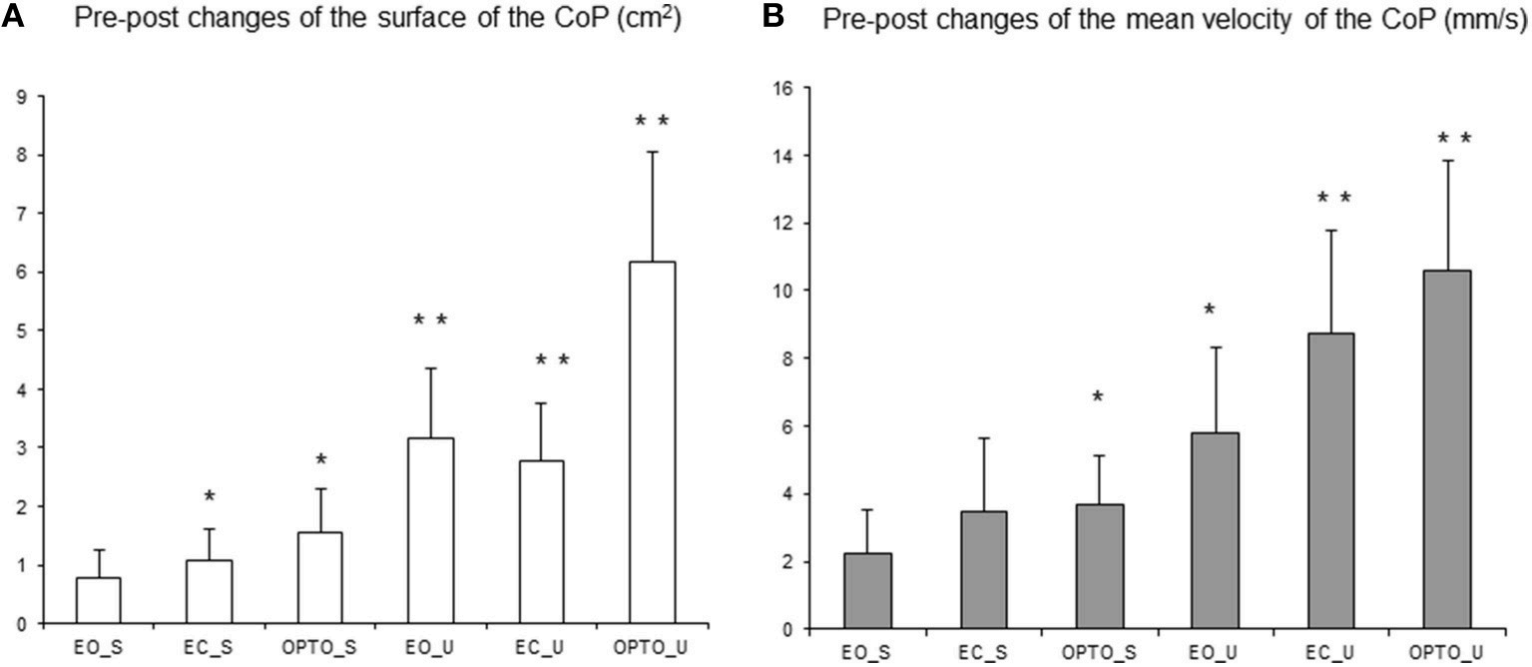
**Pre-post changes of the surface area of the CoP (in cm^**2**^) (A) and of the mean velocity (mm/s) (B) in the three visual conditions (eyes open: EO; eyes closed: EC; and with perturbed vision: OPTO) on a stable -S- or unstable -U- platform in ADHD children**. Vertical bars indicate the standard error. ^*^p < 0.05, ^**^p < 0.001.

Figure 4 summarizes changes in the surface area (Figure 4A) and mean velocity (Figure 4B) of the CoP after 4 weeks of treatment with MPH under all six experimental conditions tested. Both the mean surface area and the mean velocity of the CoP decreased after 1 month of treatment under all six conditions. The *t*-test revealed that the decrease in the surface area of the CoP after MPH treatment was significant in all but one condition (EO_S); the mean velocity of the CoP also decreased significantly in the majority of conditions tested (OPTO_S, EO_U, EC_U, and OPTO_U, respectively).

**Figure 5:**
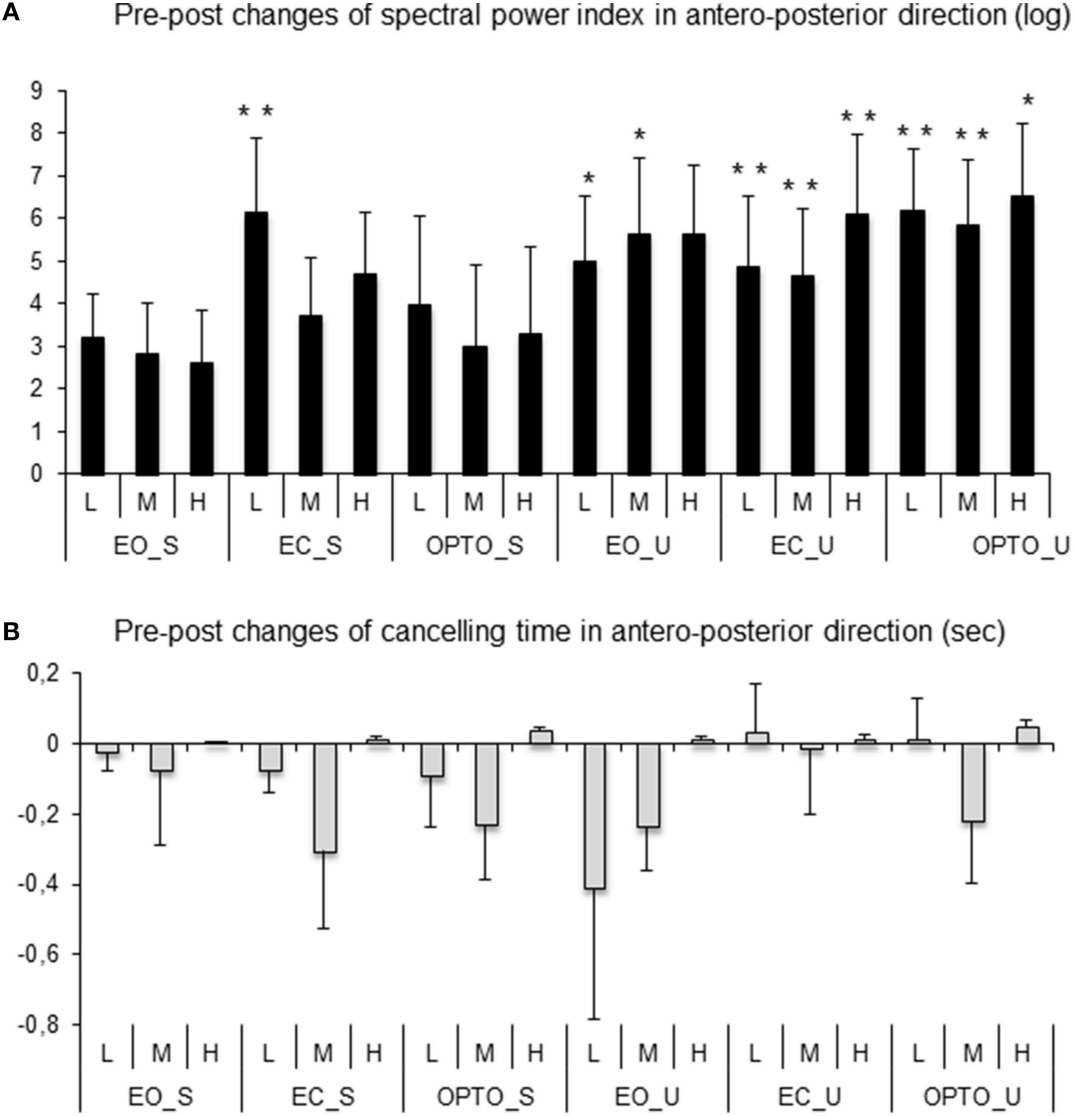
**Pre-post changes of the spectral power index (A) and canceling time (B) in the anteroposterior direction for each frequency band (L, low; M, medium; H, high) in the three visual conditions (eyes open: EO; eyes closed: EC; and with perturbed vision: OPTO) on a stable -S- or unstable -U- platform in ADHD children**. Verticals bars indicate the standard error. ^*^p < 0.05, ^**^p < 0.001.

Figure 5A reports post-treatment changes in the spectral power index for postural sway in the anteroposterior direction in all six conditions and for the three frequency bands examined. After MPH treatment, the spectral power index decreased in all conditions and for all frequency bands recorded. A *t*-test showed that the decrease in the spectral power index after MPH treatment was significant for the EC_S condition for the low frequency band and for the majority of conditions on the unstable platform (EO_U for low and medium frequencies; EC_U and OPTO_U for all three frequency bands).

After 4 weeks of MPH treatment, children with ADHD increased their canceling time (negative values in Figure 5B) under a few conditions and for low and medium frequency bands only; however these increases failed to reach statistical significance (*t*-test).

## Discussion

Although deficits in postural control in children with ADHD have been suggested by previous studies, methodological limitations have led to inconsistent results. The temporal analyses performed in our study further strengthened the hypothesis that: (i) postural control is weak in ADHD children compared sex-, age-, and IQ-matched TD children. The deficit occurred mainly for medium frequencies, pointing to a cerebellar dysfunction in ADHD. (ii) MPH significantly improved postural control, especially in unstable conditions, apparently by the more efficient processing of sensory inputs (a high-level process), as suggested by the decrease in the spectral power index, but induced no significant changes in canceling time (a low-level process).

### Poor postural control in children with ADHD

The present study confirms and expands previous findings showing poor postural control in children with ADHD. Indeed, the spatial analysis of postural measures revealed a greater surface area and velocity of the CoP in ADHD children, in line with previous studies summarized in the Introduction. The stability of the body appeared to depend on somatosensory as well as visual inputs. In our study, whatever the status of the children, stability was better on a stable platform with eyes open, while it was worse on an unstable platform when vision was perturbed by optokinetic stimulation. This finding paralleled previous observations by our group showing that healthy 4-year-old children already use both visual and somatosensory information to control their postural stability (Gouleme et al., 2014).

The deficit in postural stability was correlated with the severity of ADHD. Specifically, we observed that the surface area covered by the CoP was significantly correlated with the inattention sub-score on the ADHD-RS, on an unstable platform in both EO (*p* < 0.029) and EC conditions (*p* < 0.025). Temporal analysis of the CoP confirmed results from the static spatial analysis, but provided new and relevant information concerning the mechanisms used to compensate for the postural deficit. The frequency of body sway was significantly higher in children with ADHD. These children spent more muscular energy in controlling their stability, independently of the postural and visual conditions tested.

### Different postural strategies used by children with ADHD

Wavelet analysis of the canceling time for each frequency band provided new insights into the sensory inputs used by children with ADHD to control body sway. Our study showed that canceling time was significantly shorter in children with ADHD for low and medium frequencies, revealing a short quest time for visuovestibular and cerebellar inputs, in accordance with the hypothetical physiological significance of the different bands (Naschner, 1979; Kohen-Raz et al., 1996; Paillard et al., 2002). These visuovestibular and cerebellar inputs did not counteract the postural control deficit observed in our patients. In contrast, for the high-frequency band, children with ADHD showed significantly longer canceling times than controls, most likely because they tried to compensate for the deficit in visuovestibular and cerebellar inputs using proprioceptive information. However, the impairment in body stability did not improve in our groups of children with ADHD, considering that these inputs were very small in amplitude.

The involment of the cerebellum in motor control has been initially suggested in patients carrying cerebellar damages displaying impairments in motor control and posture. Anatomic studies also observed that the majority of the cerebellum's outputs are in relationship with the motor system. Actually, it is well known that motor commands are not initiated in the cerebellum but this latter modifies the motor commands of the descending pathways to make movements more accurate (Leigh and Zee, 2006). The cerebellum is involved in postural adjustments in order to maintain balance. Levinson suggested 25 years ago that cerebellar-vestibular testing could potentially be relevant in ADHD (Levinson, 1990). There have been strong reports of smaller cerebellar volume in children with ADHD, specifically pointing to an impairment of the cerebellar vermis. The cerebellar region identified in ADHD forms part of the dorsal and ventral attention networks (see Stoodley, 2014 for review). These works and the present behavioral study are in line with the hypothesis that children with ADHD have a dysfunctional cerebellum with poor adaptive capability (Leigh and Zee, 2006).

### Improvement of postural control after MPH treatment

The present study shows that MPH significantly improved postural stability by reducing both the surface area and the mean velocity of the CoP. Notably, we report that such changes are more relevant for postural conditions in which children have to integrate all sensory information (visual, vestibular, and somatosensory) to obtain postural stability, particularly when they are on an unstable platform, when somatosensory inputs are missing or when visual inputs are absent and/or disturbed by optokinetic stimulation. Under such conditions, cerebellar processing is needed to compensate for all these deficits and to provide efficient postural control. Peterka (2002) have also previously suggested that when sensory inputs are defective, other sub-systems compensate for the impairment by playing a more important role i.e., the sensory system is reweighted via cerebellar activity. The results of the wavelet analysis allowed us to further understand the physiological significance of the spectral power of the different frequency bands in children with ADHD before and after MPH treatment. In our study, MPH significantly improved postural control mainly under unstable conditions (decrease in the spectral power index, suggesting less consuming of body's energy), although it induced no significant changes in canceling time indicating that sensorial information via cerebellar integration are still not working correctly. These observations suggest that postural control is improved by a more efficient processing of sensory inputs (a high-level process), that could be controlled by cortical and central structures rather than a low-level process, that is controlled by more automatic systems and/or open loops which would have led to changes in canceling time. Similarly, Czerniak et al. (2013) and Ivanov et al. (2014) have suggested that the improvement of cerebellar processing in children with ADHD after MPH treatment might be the basis for their clinical recovery. ADHD children treated with MPH display larger regional volumes over the left cerebellar surface (Ivanov et al., 2014) and larger vermis sizes (Bledsoe et al., 2009) than non-treated ADHD children. Thus, MPH could help recover the integrative functions of the cerebellum and could improve postural stability. In addition, the improvement of attentional capacity after MPH treatment (clinically assessed by the ADHD-RS) was not significantly correlated to the improvement of postural parameters (see Supplementary Data). Thus, MPH could act on postural stability through indirect processes unrelated to attention. The pivotal role of the cerebellum in ADHD needs further consideration.

## Limitations

The present finding suggesting a deficiency in sensory processing by the cerebellum in children with ADHD is based on behavioral study in which cerebellar activity is indirectly observed. However, postural measures could be a promising opportunity for easily testing cerebellar performance in children with ADHD with a good acceptability. This study was conducted on a limited number of patients, and further research on a larger number of ADHD children with different doses of MPH would be useful in order to improve our understanding.

## Conclusion

In the present study, we show that postural measures could be a promising method with which to indirectly observe cerebellar performance in ADHD children. The results of this study suggest that the deficit in postural stability in children with ADHD compared to normally developing age-matched children could be due to a deficit in sensory processing by the cerebellum. MPH treatment improves postural control through adaptive strategies involving the cerebellum.

## Author contributions

MB conceptualized and designed the study. CS, EA, HP, RD selected patients. MB coordinated and supervised data collection. MB drafted the initial manuscript. MB, CS, EA, HP, RD reviewed and revised the manuscript, and approved the final manuscript as submitted.

## Funding

The authors thank the eyeBRAIN society for promoting the study. MB was supported by the Académie des Sciences, Fondation NRJ - Institut de France. The authors are also grateful to the Fondation Yves Cotrel - Institut de France for the financial support.

### Conflict of interest statement

The authors declare that the research was conducted in the absence of any commercial or financial relationships that could be construed as a potential conflict of interest.
