# Supplementary material for: Postural Instability in Children with ADHD Is Improved by Methylphenidate
Source: Front Neurosci. 2016 May 4;10:163. doi: 10.3389/fnins.2016.00163 (PMC4854903; doi:10.3389/fnins.2016.00163)
Supplement: Supplementary file 1 [file Table1.PDF]

Table 1A: Clinical characteristics of children examined

	TD	ADHD	Subsample of children with ADHD treated with MPH	
	N=38	N=38	N=26	
CLINICAL DATA				
Age (years), mean ± SEM	9.70 ± 0.43	9.82 ± 0.37	9.87 ± 0.48	
Height (cm), mean ± SEM	133 ± 40	135 ± 31		
Weight (Kg), mean ± SEM	34.2 ± 2.5	33.3 ± 3.3		
Gender (male / female)	30/8	34/4	24/2	
**ADHD-RS**				
ADHD-RS total score, mean ± SEM	4 ± 0.8	31.8 ± 1.1	38.2 ± 1.1	28.2 ± 2.1 #
ADHD-RS Inattention subscore, mean ± SEM	-	20.0 ± 0.6	19.5 ± 0.8	14.3 ± 1.0 #
ADHD-RS Hyperactivity/Impulsivity subscore, mean ± SEM	-	18.8 ± 0.8	18.7 ± 1.0	13.9 ± 1.1 #
**MPH Dose in mg/kg, mean ± SEM**	-	-	0.60 ± 0.04	
**Wechsler scale (WISC-IV) scores, mean ± SEM**				
Verbal Comprehension subscale	-	100.1 ± 2.8	99.8 ± 3.9	
Perceptual Reasoning subscale	-	94.8 ± 3.0	95.0 ± 4.1	
Working Memory subscale	-	84.1 ± 3.3	85.5 ± 3.2	
Processing Speed subscale	-	89.8 ± 2.4	90.2 ± 3.1	
Similarities test	10.36 ± 0.4	10.18 ± 0.5	10.1 ± 0.7	
Matrix reasoning test	10.54 ± 0.5	9.81 ± 0.4	10.2 ± 0.5	

[#] ADHD-RS scores after MPH treatment.
