# Supplementary figures and images for: Postural Instability in Children with ADHD Is Improved by Methylphenidate
Source: Front Neurosci. 2016 May 4;10:163. doi: 10.3389/fnins.2016.00163 (PMC4854903; doi:10.3389/fnins.2016.00163)

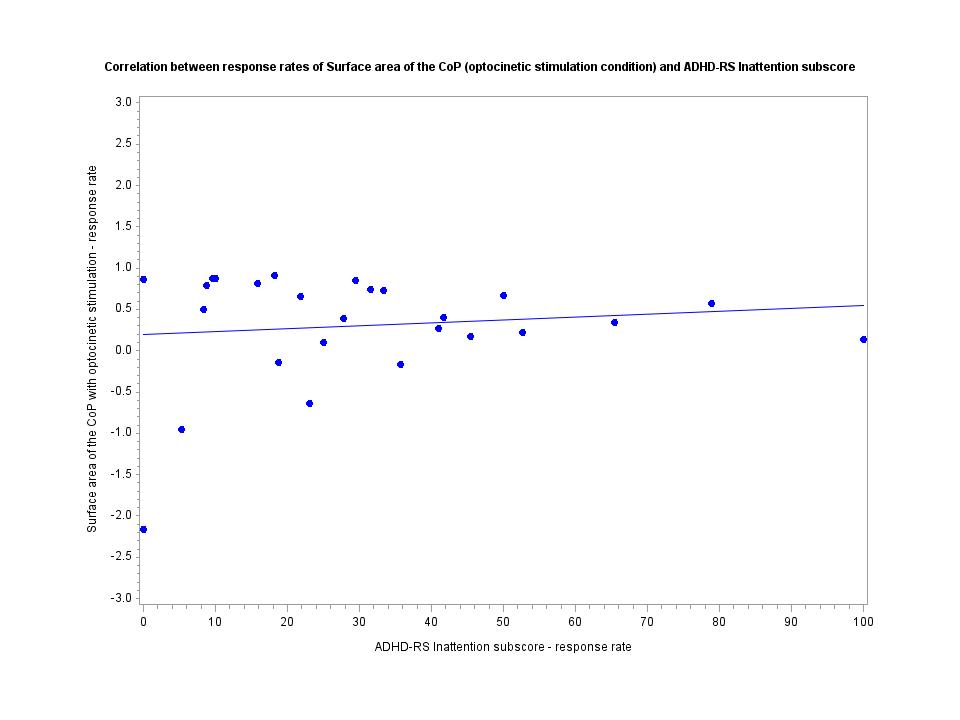

Supplement: Supplementary file 2 [file Image1.JPEG]
